# Tuning-Target-Guided Inverse Planning of Brain Tumors With Abutting Organs at Risk During Gamma Knife Stereotactic Radiosurgery

**DOI:** 10.7759/cureus.9585

**Published:** 2020-08-06

**Authors:** Qianyi Xu, Gregory Kubicek, David Mulvihill, Gary Eastwick, Howard Goldman, Alan R Turtz, Jiajin Fan, Dershan Luo

**Affiliations:** 1 Radiation Oncology, MD Anderson Cancer Center at Cooper, Mount Laurel, USA; 2 Radiation Oncology, Cooper University Hospital, Camden, USA; 3 Neurosurgery, Cooper University Hospital, Camden, USA; 4 Radiation Oncology, Inova Health System, Fairfax, USA; 5 Radiation Physics, University of Texas MD Anderson Cancer Center, Houston, USA

**Keywords:** gamma knife, inverse planning，tuning target

## Abstract

Purpose

We proposed a planning strategy that utilized tuning targets to guide GammaKnife (GK) Inverse Planning (IP) to deliver higher dose to the tumor, while keeping acceptable dose to the abutting organ at risk (OAR).

Methods

Ten patients with a large portion of brain tumor abutting the OAR previously treated with GK stereotactic radiosurgery (SRS) were selected. For each patient, multiple tuning targets were created by cropping the target contour from three-dimensional (3D) expansions of the OAR. The number of the tuning targets depended on the complexity of the planning process. To demonstrate dose sparing effect, an IP plan was generated for each tuning target after one round of optimization without shot fine-tuning. In the dose enhancement study, a more aggressive target dose was prescribed to the tuning target with a larger margin and one to two shots were filled in the region with missing dose. The resulting plans were compared to the previously approved clinical plans.

Results

For all 10 patients, a dose sparing effect was observed, i.e. both target coverage and dose to the OARs decreased when the margins of 3D expansion increased. For one patient, a margin of 6 mm was needed to decrease the maximum dose to the optical chiasm and optical nerve by 44.3% and 28.4%, respectively. For the other nine patients, the mean dropping rate of V12Gyto brain stem were 28.2% and 59.5% for tuning targets of 1 and 2 mm margins, respectively.

In the dose enhancement study, the tuning-target-guided plans were hotter than the approved treatment plans, while keeping similar dose to the OARs. The mean of the treatment and enhancement dose was 15.6 ± 2.2 Gy and 18.5 ± 3.2 Gy, respectively. The mean coverage of the target by prescription dose was slightly higher in the enhancement plans (96.9 ± 2.6% vs 96.3 ± 3.6%), whereas the mean coverage of the enhancement dose was 20.1% higher in the enhancement plans (89.6 ± 9.0% vs 74.6 ± 19.9%).

Conclusions

We demonstrated that an inverse planning strategy could facilitate target dose enhancement for challenging GK cases while keeping acceptable OAR dose.

## Introduction

Gamma Knife (GK) is an effective platform to deliver stereotactic radiosurgery (SRS) to intracranial tumors. Depending on the model of the system, narrow beams from 192 or 201 Cobalt-60 sources are converged to the isocenter as a shot in the treatment planning system (the Leksell GammaPlan®, Elekta AB, Stockholm, Sweden). Except for very small lesions and those with a shape easily covered by a single shot with a strategic isodose line, multiple shots are typically needed to paint a desired target dose and each shot contributes a portion of the total dose. Forward planning (FP) has been the choice since the launch of GK. The planner manually places the shots and adjusts shot parameters to make a satisfactory plan. Those parameters include the number and size of the shots, their locations, weights and collimator settings, etc. The quality of a manual GK plan highly depends on the planner’s experience. The planning process is tedious and time consuming, especially for large targets with many shots.

The Inverse Planning (IP) technique for GK SRS was proposed by multiple groups [1-4] and was not commercially available until Elekta released the GammaPlan® version 10.0 in 2010 [5]. The IP feature allowed the users to iteratively optimize desired target dose. It started from assigning planning parameters by the planner, e.g., isodose line, coverage, selectivity, low dose spillage and beam-on time, etc. The GammaPlan® iteratively searched for the planning goal and the dose was displayed and updated in real-time. The planner could pause optimization at any time and adjust the optimization parameters for the next round. The IP technique has been shown overall easy to operate with reasonable plan quality and improved planning efficiency, especially for large targets [6-8].

Some issues arose when there was an organ at risk (OAR) adjacent to the target or, in a worse scenario, abutting the target. In the GammaPlan® (version 11.1.1), it is difficult to impose a constraint on volume limit or maximum dose to the OARs during optimization. Manual fine-tuning, which included dynamic shaping, of the shots was necessary to reduce the dose to the OAR before finalizing a plan. The fine-tuning process became more challenging for some sizable targets with a larger portion of their volume abutting the OARs. To reduce the dose to the OARs, the planner had to change the location of the nearby shots, as well as the shape and weight of the shots. Compared to planning for a small lesion, the planning process for a large lesion with OARs in its vicinity is more challenging. First, many more shots were often used for larger targets and fine-tuning process became very tedious. Second, when shots were manually pushed away from the OAR boundary, the dose in the distal side of the target became compromised, leading to another round of shot fine-tuning. Lastly, GK planning needed to be done timely, as the patient fixated with a frame was waiting for the start of treatment. To prioritize the dose constraints to the OAR over the target coverage, physicians often had to lower the prescription dose for the entire target.

In this study, we proposed an efficient and effective planning strategy to facilitate IP for these challenging cases. A tuning planning target was first constructed from the target after cropping the three-dimensional (3D) expansion of the abutting OARs with a margin and whereby used to guide the GammaPlan® to optimize the intended dose on the tuning target and avoid high dose to the OARs. The structure of the manuscript was organized as following: 1) to demonstrate the effectiveness of the tuning structures to spare the dose to OARs under an IP setting and 2) to further enhance the dose to the distal part of targets utilizing tuning structures, while keeping OAR dose similar to the previously approved treatment plans.

## Materials and methods

Patient selection

**Figure 1:**
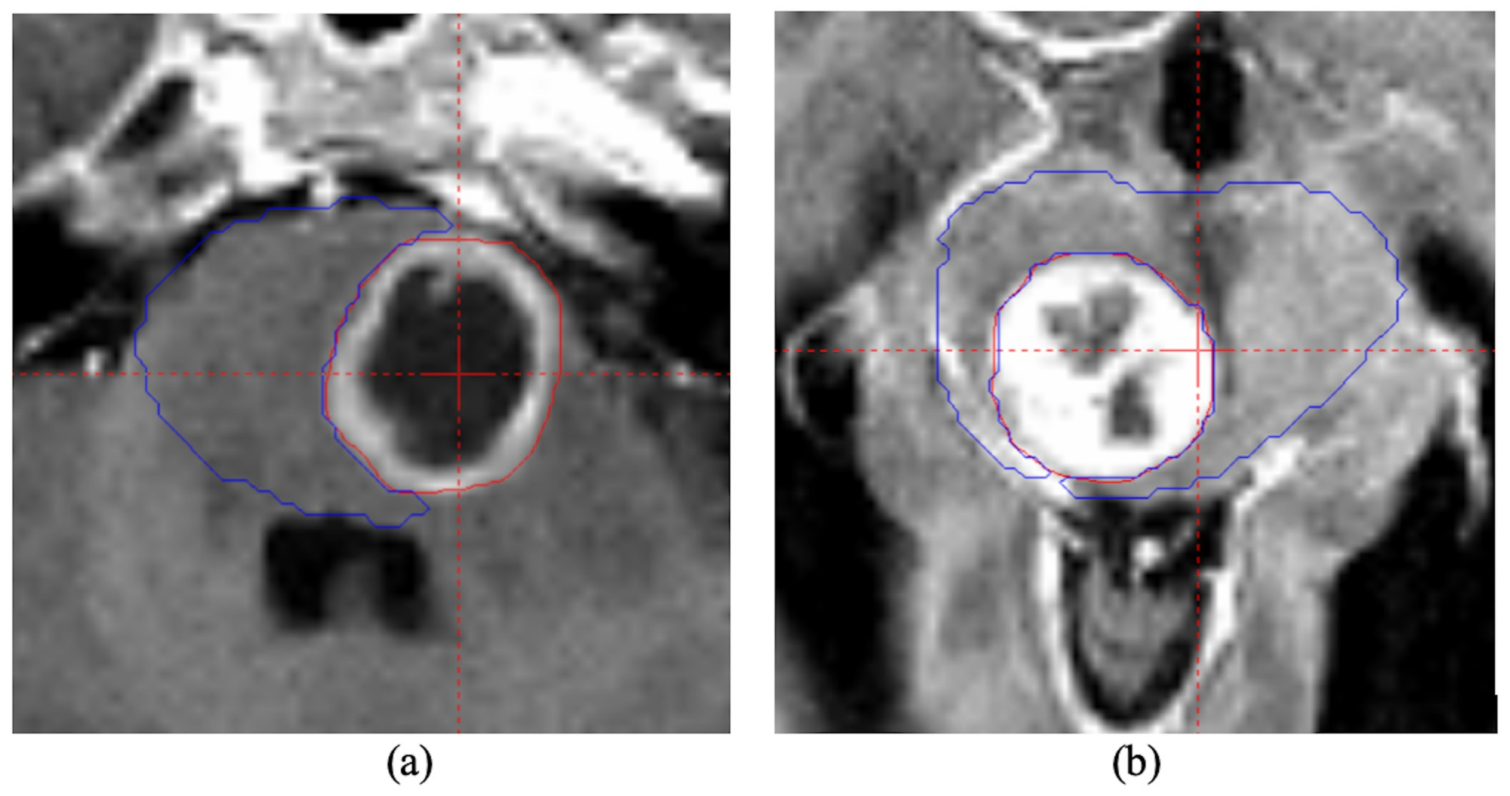
The axial slice of T1-weighted Magnetization Prepared RApid Gradient-Echo (MP RAGE) MR image post contrast for two patients. The red line indicated the tumor drawn by the physician and the blue line was the brain stem. They were the patient 3 (a) and 5 (b) in Table 1.

Ten patients previously treated with GK SRS were selected in the study with a criterion of a large portion of tumor volume abutting the OAR (Table 1). The volumes of the tumors were relatively large, with the mean and standard deviation (std.) of 7.3 ± 5.3 cc. The dimension (the maximum size in one direction) of the tumors ranged from 2.2 cm to 3.8 cm (2.8 ± 0.4 cm). One of the patients was diagnosed with right temporal meningioma abutting the optical apparatus and the other nine patients were brain metastasis or meningioma abutting the brain stem. Figure 1 showed the screenshots of the axial magnetic resonance (MR) image for two patients (patient 3 and 5 in Table 1). To protect the OARs, the coverage and prescription dose (15.6 ± 2.2 Gy) were compromised in the delivered treatment plans.

**Table 1:** Patient tumor characteristic. OAR: Organ at Risk

Patient	Diagnosis	Maximum dimension (cm)	Volume (cc)	OAR	Dose (Gy)	# of Tuning Targets
1	meningioma	2.9	9.4	Optical apparatus	13	6
2	metastases	2.2	2.6	Brain stem	18	3
3	metastases	2.8	6.6	Brain stem	15	2
4	metastases	2.8	8	Brain stem	18	4
5	metastases	3.8	20.1	Brain stem	15	2
6	metastases	2.6	6.1	Brain stem	18	3
7	meningioma	3.2	5.7	Brain stem	13	2
8	meningioma	2.5	1.7	Brain stem	14	2
9	metastases	2.5	2.5	Brain stem	18	2
10	meningioma	2.9	10.1	Brain stem	14	2

Planning process based on tuning structures

The IP feature in the GammaPlan® was well introduced by Schlesinger et al. [5]. The following is a brief description of the planning steps and typical planning parameters used in our study. Before optimization started, an initial set of shots were needed as starting shots. This could be achieved through either manual or automatic filling by the GammaPlan®. Due to relatively large size of the tumors, we adopted automatic filling by choosing smaller collimator size setting. This would generate an excessive number of starting shots to ensure a sufficiently large search space. The length of treatment time was highly related to the number of the shots. However, the Beam-on setting was able to ensure acceptable treatment time (Beam-on setting of 0.1 was used for all the cases). The Coverage and Selectivity settings were conflicting to each other. Higher coverage led to less conformal target dose and vice versa. For all the cases, the Coverage setting of 0.68 and the Selectivity setting of 0.32 were chosen based on our experience. The Gradient Index (GI) was used to control the low dose falloff (GI setting of 0.19 was used for all the cases). The definition of these parameters could be found in Schlesinger et al. [5].

To demonstrate the dose sparing effect, multiple tuning targets were created before planning started. As current GammaPlan® was not capable of contour processing, the MR images and structure set were sent to a third-party software (VelocityAI®, Varian, CA, USA) first. Tuning targets were generated by cropping the target contour from the 3D expansion of the OAR. The number of the tuning targets depended on the complexity of the planning process (Table 1). More tuning targets (with increasing margins) were used for a more complex planning process. For the most complex case (Patient 1), six tuning targets were generated with margins from 1 mm to 6 mm. The number of the tuning targets corresponded to the different sizes of margins added to the OAR expansion. When contour processing was done, the tuning targets were sent back to the GammaPlan® for planning.

For each tuning target, an IP plan was generated with the same optimization parameters to evaluate dose sparing effect. Only one round of optimization was allowed for each tuning target and no shots were modified nor were additional shots added. A general dosimetry guideline was adopted for dose evaluation. For the brain stem, we required V12Gy < 0.1 cc for benign tumors and V12Gy < 1 cc for malignant tumors. For the optical apparatus, we used the criterion of maximum dose < 10 Gy, which was defined as the dose received by a small volume (1 mm3). Once optimization started, we waited until the objective function converged and stabilized (within about 2-3 minutes). The tuning targets were used solely for optimization purpose and the original tumor volume was used for dosimetry evaluation.

Dose enhancement study

As demonstrated, the OAR doses were lowered by using the tuning targets during optimization. This led to a dose enhancement study investigating the delivery of higher dose to the tumor at the distal side, while maintaining an acceptable OAR dose. The original and enhanced prescription doses were listed in Table 2 in the Results section. To investigate the effect of dose enhancement, the optimized plans, previously unaltered after optimization was completed, were fine-tuned with one to two shots to fill in the region where anatomy changed abruptly and missing dose typically occurred, and the prescription dose was raised to achieve comparable OAR dose. The dosimetric parameters in the dose enhancement plans were compared to the previously delivered treatment plans.

## Results

**Figure 2:**
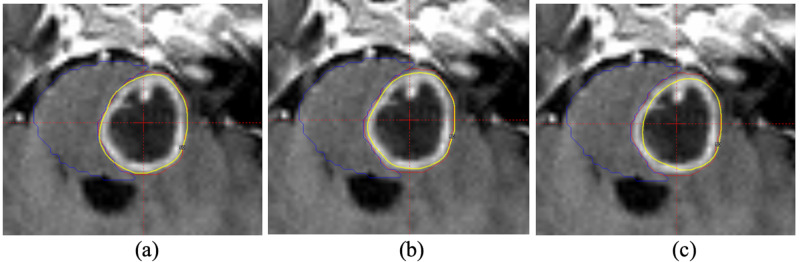
The volume sparing effect was shown on MR for patient 3. Three tuning targets were used (0 mm (a), 1 mm (b) and 2 mm (c)) and the corresponding prescription isodose lines (yellow) were plotted. The red contour was the target drawn by the physician and the blue contour was brain stem.

The optimized plans were quantitatively and visually evaluated for all 10 patients. The margins used for the tuning targets for all patients were from 2 mm to 6 mm (mean of 2.9 ± 1.3 mm). The results from the patient 3 (Figure 1a) were shown in Figure 2 to demonstrate the dose sparing effect. The 0-mm margin meant that original target was used for optimization (Figure 2a). From Figure 2b to Figure 2c, a margin from 1 to 2 mm was used to generate the tuning targets. The dose sparing effect was clearly observed: the larger the margin, better sparing of the OAR. The target coverage for the plans from Figure 2a to Figure 2c was 95%, 88% and 78% and the corresponding V12Gy for the brain stem was 1.94 cc, 0.89 and 0.2 cc. Meanwhile, the dose in the distal right side of the tumor was still very conformal. Without tuning targets, the dose conformality in the distal side of the tumor could be compromised due to manual pushing of the shots away from the OAR. It was worth noting that all three plans were generated after one round of optimization without shot fine-tuning.

**Figure 3:**
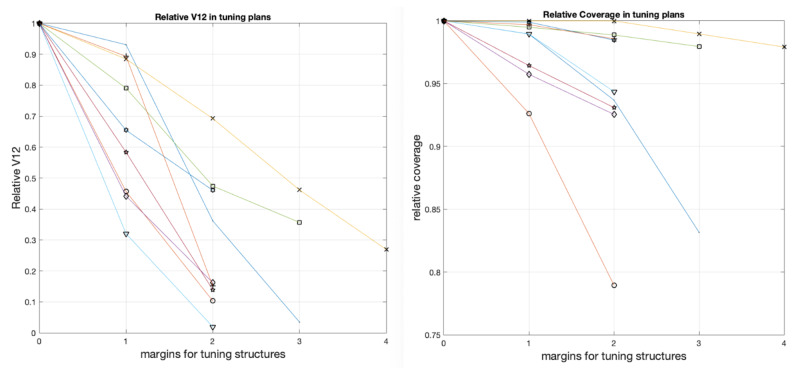
The brain stem dose (a) and target coverage (b) with different tuning targets for nine targets abutting the brain stem. The relative V12 and coverage were normalized so that the V12 and coverage of the 0 mm-margin plan were unity.

**Figure 4:**
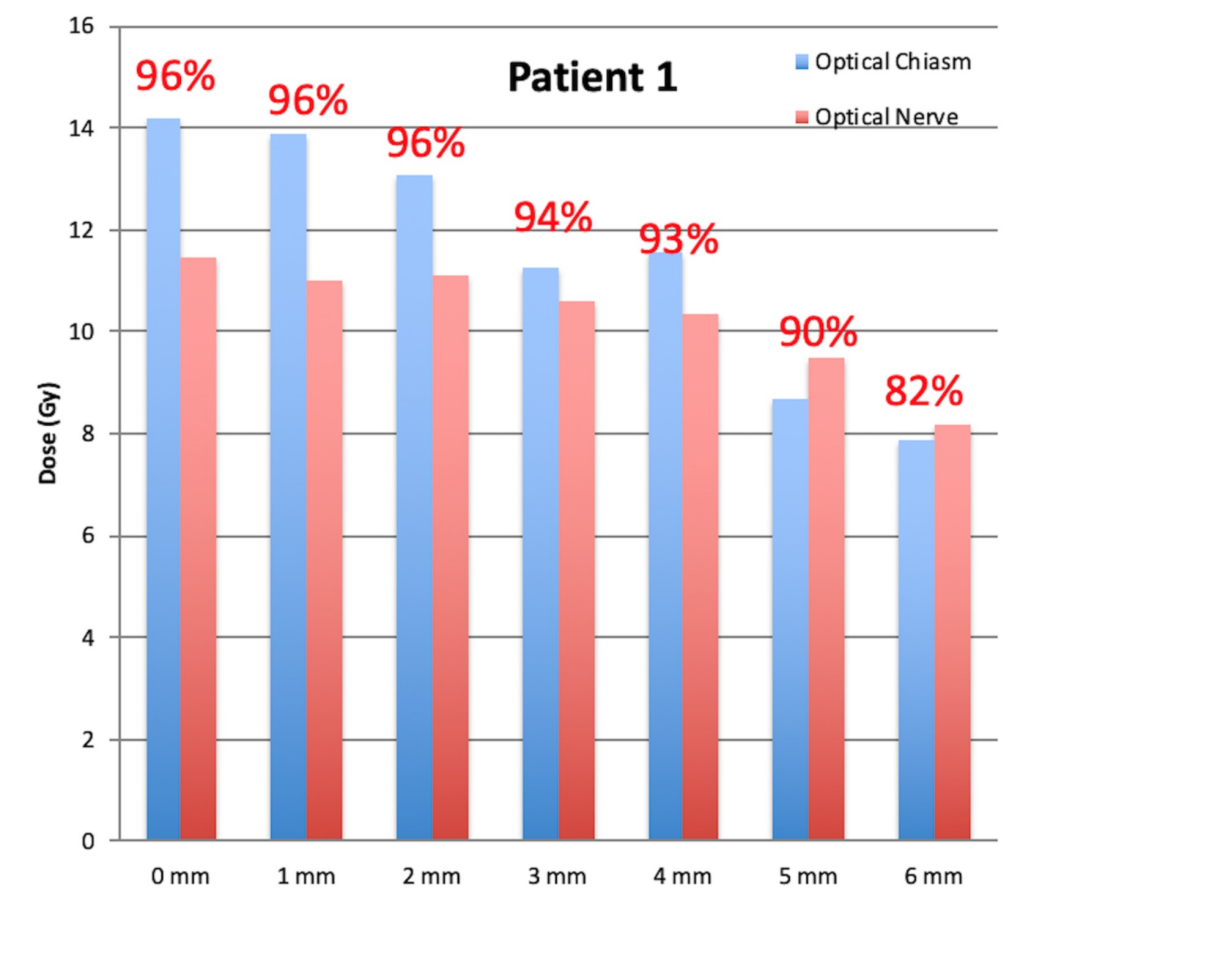
The maximum dose to the optical apparatus and target coverage with different tuning targets for patient 1. The target coverage by different margin was labeled on the top of the bars correspondingly.

For all nine targets abutting the brain stem, the relative V12Gy of the brain stem and coverage for all the tuning plans were plotted in Figure 3, using the V12Gy and coverage in the plan of 0 mm margin as the reference. A trend was observed that both clinical target coverage and dose to the brain stem dropped when margins increased for tuning targets. For the target abutting the optical apparatus (patient 1), the maximum dose to the optical chiasm and nerve vs target coverage were plotted in Figure 4 and similar dropping trend was observed. However, the volume limit in Figure 3a dropped significantly faster than the maximum dose to chiasm and optical nerve in Figure 4. For patient 1, a margin of 6 mm was needed to drop the maximum dose to the optical chiasm and optical nerve by 44.3% and 28.4%, respectively, comparing to the plan without margin. For the other nine patients, the mean dropping rates of V12Gy were 28.2% and 59.5% for tuning structure with 1 and 2 mm margins. This was explainable in that the tuning target was constructed to cool down a portion of the tumor volume close to the OARs and volume sparing was in favor consequently. The maximum dose was determined by the local shots and might not be as sensitive as the volume sparing.

**Figure 5:**
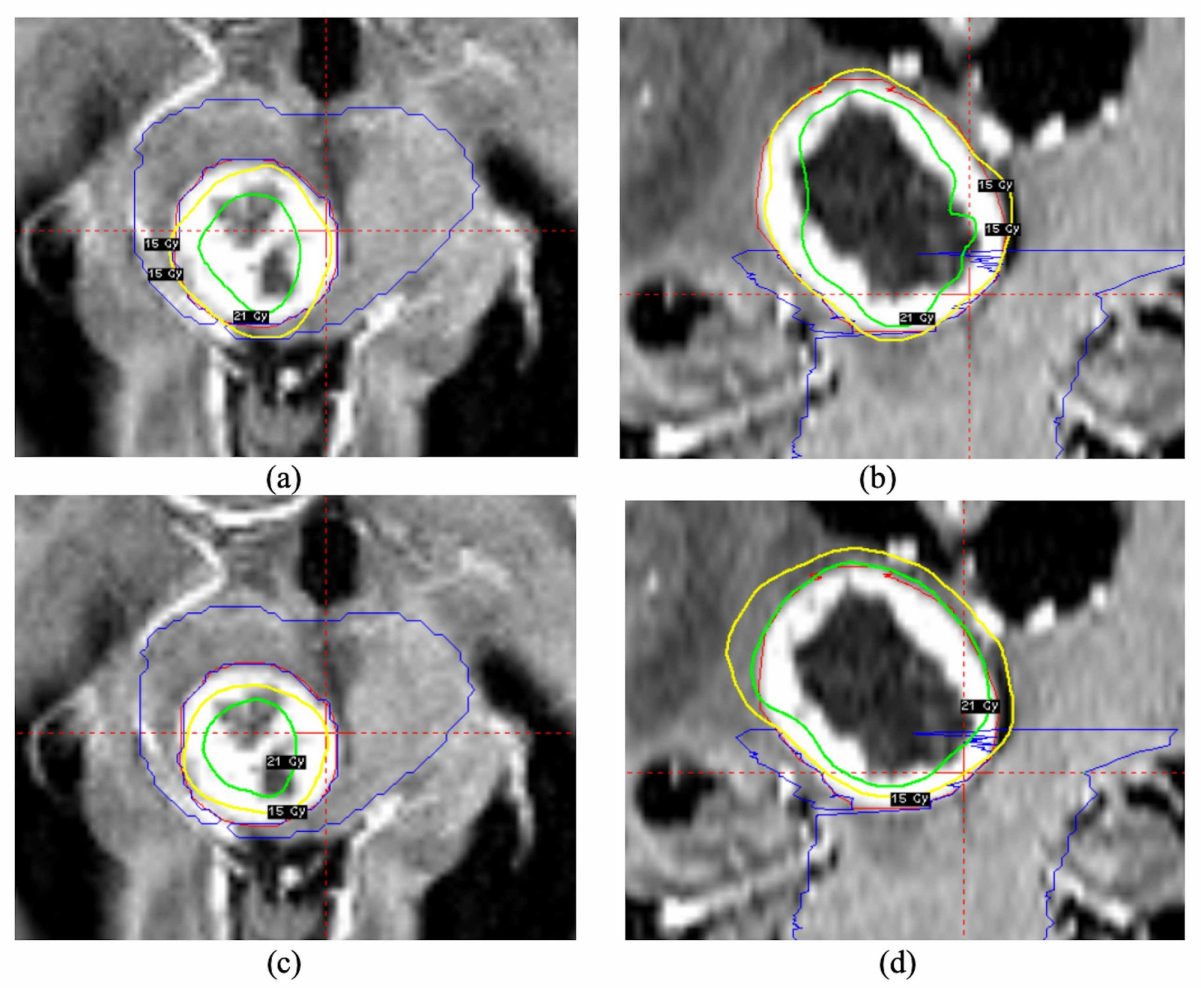
The comparison of the clinical and dose enhancement plans for the patient 5. The clinical plan was shown in the axial plane (a) and coronal plane (b). The dose enhancement plan was shown in the axial plane (c) and coronal plane (d).

In the dose enhancement study, the clinical plans and dose enhanced plans were compared quantitatively and visually for all the patients. The plans from the patient (patient 5 in Table 2) were shown in Figure 5 with the original prescription dose (15 Gy) and enhanced dose (21 Gy). The inner isodose line was 21 Gy and the outer isodose line was 15 Gy. In the axial planes, no significant dosimetric difference was observed between the treatment (Figure 5a) and enhanced plan (Figure 5c). However, for the distal part of the tumor (the coronal view of treatment dose in Figure 5b and enhanced dose in Figure 5d), greater dosimetric difference was noticed. In the treatment plan (Figure 5b), the majority of the distal tumor was covered by 15 Gy isodose line, whereas in the enhanced plan, the distal part of the tumor was well covered by 21 Gy. For all patients, the target coverage and dose to the OARs were listed in Table 2 for the treatment and enhancement plans. For some enhancement plans, we slightly adjusted the prescription dose to match the dose to the OARs in both plans. The mean of the treatment and enhancement dose was 15.6 ± 2.2 Gy and 18.5 ± 3.2 Gy, respectively. The coverage of prescription dose was slightly higher in the enhancement plans (96.9 ± 2.6% vs 96.3 ± 3.6%), whereas the mean coverage of the enhancement dose was 20.1% higher in the enhancement plans (89.6 ± 9.0% vs 74.6 ± 19.9%). For patient 1, the maximum dose to the optical chiasm and nerve was 9.4 Gy and 9.1 Gy in the enhancement plan and 9.8 Gy and 8.9 Gy in the treatment plan. For the other nine patients, the V12Gy of the brain stem was 0.45 ± 0.59 cc in the enhancement plans and 0.57 ± 0.76 cc in the treatment plans.

**Table 2:** Dosimetry comparison between treated and dose enhancement plans. Cov_T_O and Cov_T_E were the coverage of the treatment dose in the treatment and enhancement plans. Cov_E_O and Cov_E_T were the coverage of the enhancement dose in the treatment and enhancement plans. V12Gy_T and V12Gy_E were the V12Gy of the brain stem in the treatment and enhancement plans. The dose before parentheses was delivered dose and the dose in parentheses was used for dose enhancement study.

Patient	Cov_T_O	Cov_T_E	Cov_E_O	Cov_E_E	V12Gy_T	V12Gy_E	Dose (Gy)
1	87.0%	91.4%	72.6%	77.1%			13 (15)
2	98.4%	98.4%	87.5%	94.3%	0.41 cc	0.50 cc	18 (21)
3	95.9%	94.8%	40.7%	74.3%	1.79 cc	1.81 cc	15 (21)
4	96.4%	98.7%	77.7%	92.1%	0.14 cc	0.08 cc	18 (21)
5	94.8%	94.0%	37.0%	79.4%	2.00 cc	0.99 cc	15 (21)
6	99.1%	99.6%	81.1%	95.6%	0.32 cc	0.25 cc	18 (21)
7	95.7%	97.4%	86.2%	93.3%	0.11 cc	0.10 cc	13 (14)
8	97.5%	98.3%	94.2%	96.9%	0.10 cc	0.10 cc	14 (15)
9	99.6%	98.2%	78.9%	97.3%	0.12 cc	0.11 cc	18 (21)
10	98.5%	97.7%	90.0%	95.2%	0.10 cc	0.10 cc	14 (15)

## Discussion

Historically, manual planning has been the choice since the launch of GK SRS. The IP feature was introduced as an additional planning option besides manual planning in the GammaPlan® version 10. The IP for GK SRS has not yet gained the same popularity as its counterpart for conventional IMRT planning, due to user habit and no need for small lesions, etc. The initial performance of the GammaPlan® version 10 was evaluated by Schlesinger et al. [5]. After the GammaPlan® was upgraded to version 11.1.1, we performed a retrospective planning study to compare the treated manual plans with the replanned inverse plans [8]. For tumors with size > 1 cm, we found that IP plans outperformed the manual plans. Because the tumor size in the study was relatively large, IP would be a good choice for planning due to its efficiency. With further help from tuning targets, IP could generate plans to spare OAR doses with different margins.

Several studies have reported positive correlation between the target dose and Local Control (LC) for brain metastases SRS [9-11]. For 261 metastases treated in 119 patients, Shiau et al. found longer freedom from progression and better LC in 145 lesions receiving at least 18 Gy in a single fraction SRS [10]. In another study by Vogelbaum et al., a significantly lower risk of local failure was found for target dose of 24 Gy than either 15 or 18 Gy for 375 metastases [11]. The one-year LC was 85% for 24 Gy dose group vs < 50% for 15 and 18 Gy group. In a recent study, Abraham et al. reviewed a total of 612 non-small cell lung cancer brain metastasis treated with SRS and found the target volume receiving more than 32 Gy was an independent predictor of LC [9]. For the entire cohort, one-year LC was 89% for the tumors with V32Gy > 24% of the total volume, vs LC of 67% for the tumors receiving V32Gy < 24% of the total volume. Similar findings were reported in other literatures [12, 13]. A very conformal and high target dose was desired to improve LC and achieve therapeutic goals. However, it is unfavorable if a high target dose also causes symptomatic radiation necrosis. For the cases in this study, the prescribed dose was at the lower end, as the OAR dose was prioritized. With the tuning targets, we believed that the distal side of the tumor could be optimized to receive higher therapeutic dose, while maintaining acceptable OAR dose and eventually patients would achieve better LC. Note that the proximal side of the tumor in the enhancement study received the same dose as the clinical plan.

The current study could be further improved in the future. First, there was no contour processing in current version of GammaPlan®. The Digital Imaging and Communications in Medicine (DICOM®) images and targets had to be sent to a third-party software to be processed and sent back to the GammaPlan®. This was not streamlined in GK workflow. We are hopeful that contour processing will be available in the future release of the GammaPlan®. Second, in this study, we demonstrated the correlation between the size of margins for the tuning target and dose to the OARs. In practice, the choice of the margin was still empirical depending on the complexity of planning and user experience. It could be improved by allowing the user to specify the volume and the extent of the OAR to be spared and, from such input, the Treatment Planning System (TPS) to calculate the margin for the tuning target. This would eliminate the need to estimate the margin based on the shape and relative locations of the target and OARs. Eventually, like other TPS, the volume limit should be incorporated into optimization process as a penalty function.

## Conclusions

An inverse planning strategy without tedious shot fine-tuning was proposed for challenging GK cases. Different dose sparing effects were observed when margins were applied to the tuning structures. The proposed method could also facilitate target dose enhancement while keeping acceptable OAR dose.
